# Comparative Transcriptomic Profiling to Understand Pre- and Post-Ripening Hormonal Regulations and Anthocyanin Biosynthesis in Early Ripening Apple Fruit

**DOI:** 10.3390/molecules23081908

**Published:** 2018-07-31

**Authors:** Jakaria Chowdhury Onik, Xiaojia Hu, Qiong Lin, Zhidong Wang

**Affiliations:** Institute of Food Science and Technology, Chinese Academy of Agricultural Sciences/Key Laboratory of Agro-products Quality and Safety Control in Storage and Transport Process, Ministry of Agriculture and Rural Affairs, Beijing 100193, China; j.conik@yahoo.com (J.C.O.); huxiaojia2009@foxmail.com (X.H.); wangzhidong@caas.cn (Z.W.)

**Keywords:** apple fruit, hormone, anthocyanin, pre-ripening, post-ripening, RNA-seq

## Abstract

The ‘Hongyu’ apple is an early ripening apple cultivar and usually used for fresh marketing. Due to the short ripening period, most of the fruit are harvested at the commercial maturity stage for proper marketing distribution and a longer shelf life. Fruit ripening involves delicate changes to its metabolic and physiological traits through well-organized synchronization of several hormones and regulatory steps. A clear understanding of these hormonal alterations is crucial for extending the period from commercial to physiological ripening. This study was intended to clarify the hormonal alterations and anthocyanin biosynthesis process prior to and immediate after, the harvesting of apple fruit considering the commercial maturity stage. Fruits harvested at 120 Days after flowering (DAF) (HY_4th) was considered as commercially ripened, 110 DAF (HY_3rd) as pre-ripening and 120 DAF followed by five days storage at 20 °C (HY_20 °C_5) as post-ripening samples. Three different stages of fruit were used for transcriptome assembly using RNA-Seq. Results revealed 9187 differentially expressed genes (DEGs) in the post-ripening samples, which was comparatively lower (922 DEGs) in the pre-ripening fruits. DEGs were subjected to Gene Ontology analysis and 31 categories were significantly enriched in the groups ‘biological process,’ ‘molecular function’ and ‘cellular component.’ The DEGs were involved in hormonal signaling pathways like ethylene, abscisic acid (ABA), auxin, gibberellin (GA), brassinosteroid (BR) and anthocyanin biosynthesis pathways such as *PAL*, *4CL*, *CHI*, *DFR*, *F3H*, *UFGT*. Several transcription factors like the MADS-box gene, *MYB*, *bHLH*, *NAC*, *WRKY* and *HSF* were differentially expressed between the pre- and post-ripening fruits. Selected DEGs were subjected to gene expression analysis using quantitative RT-PCR (qRT-PCR) and the results were consistent with those of RNA-Seq. Our data suggested that in addition to ethylene, ABA and other hormones also play key roles in regulating apple fruit ripening and may interact with the ethylene signaling process. Additionally, our data provided an exhibition of the expression pattern of genes in the anthocyanin biosynthesis pathway.

## 1. Introduction

The apple is one of the important fruit crops all over the world, receiving attention for its delicious taste and considerable nutritive value. Apple fruit endures rapid ethylene production which triggers a dramatic change in many important quality attributes, including colors, sugars-acids ratio and others, during development and ripening [1]. Previous studies have confirmed a critical regulatory role of ethylene in fruit softening by cell wall degradation [2], biosynthesis of volatiles compounds providing aroma [3] and loss of green color by the accumulation of anthocyanin [4].

Plant hormones are reportedly considered to be closely linked with fruit development as well as ripening [5]. By the response of different hormones, the significant ripening regulations seem to be controlled primarily by ethylene and ABA [6,7]. Considering the crucial role of ethylene in climacteric fruit ripening, it remains the most researched hormone [8]. Previous studies on tomatoes provide a background of ethylene signal transduction and response [9]. Involvement of several genes in ethylene biosynthesis process is well noted. Rate limiting genes such as 1-aminocyclopropane-1-carboxylic acid synthase (ACS) and 1-aminocyclopropane-1-carboxylic acid oxidase (ACO) were primarily regulated by RIN and MADS-box gene family [10]. The silencing of *MdMADS8* resulted in a significant decrease of *ACS1* and *ACO1* and has been observed in the transgenic apple line, which indicated a strong correlation of MADS-box genes with ethylene biosynthesis [2].

However, understanding the roles of other hormones other than ethylene during fruit ripening is limited. A transcriptome study with different ripening stages of tomato fruits has revealed that next to ethylene, auxin-related genes were the most represented in hormone response category [11]. Interactive relations of ethylene and indole-3-acetic acid (IAA) in climacteric fruits ripening has been noted in several studies [11]. A declination of free auxin level (indole-3-acetic acid, IAA) before the ripening has been observed in both climacteric (tomato) and non-climacteric (pepper, strawberry) fruits and presumed this reduction caused by the increase of IAA-Asp (IAA-aspartic acid), a conjugated form of free auxin [12,13]. In tomato and peach fruits, ethylene biosynthesis genes (*ACS2*, *ACS4* and *ACO1*) and ethylene signaling genes (*ETRs* and *ERFs* etc.) were up-regulated by auxin [14,15]. In the tomato, two IAA-amino synthase genes (*GH3*) exhibited a pattern of expression associated with ripening, which catalyzed the reaction of IAA-Asp (IAA-aspartic acid) conjugation [16]. Overexpression of pepper *GH3* gene in tomato fruits directed towards anticipatory ripening process [17] which indicated the participation of conjugated form of IAA rather than only IAA, may contribute to the temporal regulation of ripening [13].

The profound roles of auxin in the ripening of some non-climacteric fruits have been well-studied; whereas little effort given to investigate the possible roles of other plant hormones as gibberellin (GA). However, an external application of GA3 on ripening strawberry fruits has significantly retarded red color development [18] and altered the expression of genes participate in cell enlargement process [19]. Abscisic acid (ABA) is a well-known phytohormone that takes part in several means of growth and development and stress response in plants [20]. Studies have reported the role of ABA in promoting sugar accumulation in fleshy fruits [21] and regulating the ripening of climacteric fruits. The down-regulation of cell wall degrading genes such as polygalacturonase (*PG*) was observed in tomato fruit due to the suppression of *NCED1* (9-cis-epoxycarotenoid dioxygenase), which catalyzed the key steps in ABA biosynthesis resulting in an increase in firmness and to extend shelf-life [22]. Although several studies on brassinosteroid (BR) have been conducted, its role in fruit ripening is still indistinct.

Anthocyanins are secondary metabolites, which play a substantial part in pigmentation with many health benefits as an antioxidant and with anti-tumor properties [23]. The biosynthesis and accumulation of anthocyanins are determined by metabolic networks correlated with the expression of several genes and regulatory factors [24]. Characterization of key genes involved in the anthocyanin metabolic process has been studied by several researchers and has revealed structure genes in biosynthesis. Direct involvement of the genes encoding chalcone synthase (*CHS*) [25], chalcone isomerase (*CHI*) [26], dihydroflavonol-4-reductase (*DFR*) [27] and glucosyltransferase (*UFGT*) [28] has been found in the anthocyanin biosynthesis process. The expression patterns of these genes are differentially exhibited with different fruit developmental stages and ripening in different species [29]. Moreover, several transcription factors have been identified which are functional for regulating the anthocyanin biosynthesis process through interaction with structure genes characterized by genetic analysis in different fruits as strawberry and grape [30,31].

Overall, fruit ripening initiation is mainly characterized by ethylene synthesis, color changes and cell wall dynamics [32,33], whereas other ripening characteristics have received less attention and are yet to be investigated. McAtee et al. [6] mentioned that individual ripening processes themselves might be under specific hormonal control. Reports on the interaction between hormones at several levels above biological processes are limited and unclear. However, due to recent advances in using transcriptomic, proteomic and metabolomic analytical tools, significant progress has been achieved in the characterization of hormonal responses [34,35,36,37,38] which have been directed to the identification of the distinct expression of genes associated with hormonal response across plant species [22,39,40]. Total transcriptome analysis of fruits before the onset of ripening and immediately after ripening considering commercial maturity would be helpful to understand the changes and respective regulations more clearly. Our study involved a comparative transcriptome analysis in facilitating the understanding of ripening hormonal control in early ripening apple fruit. Several genes associated with plant hormones, the anthocyanin biosynthesis process and a few transcription factors were differentially expressed during fruit ripening. Also, the expression of selected differentially expressed genes was confirmed by quantitative RT-PCR (qRT-PCR).

## 2. Results

### 2.1. Changes in Organic Acid and Sugar Content in Pre- and Post-Ripening Apple

**Figure 1:**
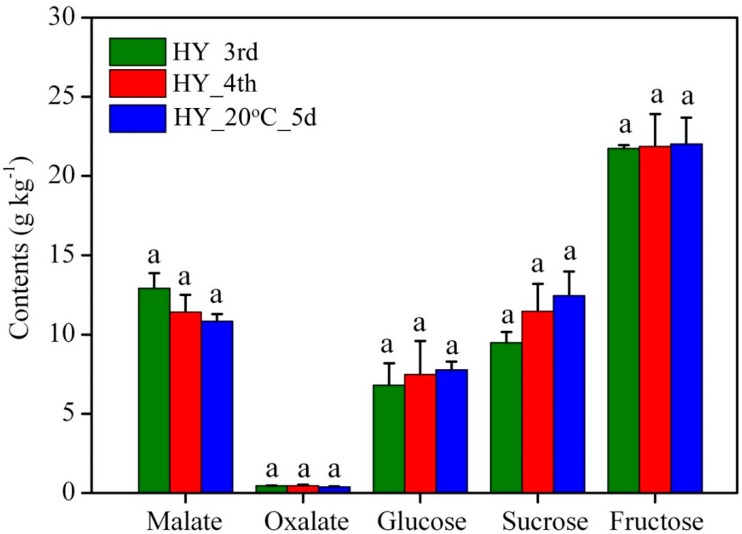
Variation of malate, oxalate, glucose, sucrose and fructose content in pre-ripening (HY_3rd), ripening (HY_4th) and post-ripening (HY_20 °C_5) ‘Hongyu’ Apple fruit. The error bars represent the standard errors. The significant differences were calculated at the 0.05 level.

The main organic acids and sugars were measured at different stages of the ‘Hongyu’ apple. Two organic acids, including malate and oxalate, were measured in this analysis (Figure 1). From the results, it can be seen that malate was the most prominent organic acid in apple fruit. The oxalate contents were below 0.5 g kg^−1^, while the malate contents varied from 10 to 13 g kg^−1^. The highest malate content was observed in HY_3rd samples (12.92 g kg^−1^) which showed a decreasing trend in the HY_4th (11.41 g kg^−1^) and HY_20 °C_5 samples (10.83 g kg^−1^). Glucose, fructose and sucrose were the main sugars in apple fruit, in which fructose content was prominent. According to the results, glucose and sucrose showed an increasing pattern of ripening (HY_4th) and post-ripening (HY_20 °C_5) samples. The changes observed in this study agreed well with previous studies that also used the date of full bloom as a reference point [41,42,43].

### 2.2. RNA-seq and Deferential Gene Expression Analysis

Pre- and post-ripening apple samples were subjected to RNA-seq analysis. Fruits of 110 DAF denoted as HY_3rd, 120 DAF denoted as HY_4th and stored for 5 d at 20 °C indicated as HY_20 °C_5 groups in transcriptome analysis. cDNA libraries were constructed from the total RNA of pre-ripening and post-ripening apple fruits. With Illumina sequencing technology through pair-end reading, we got more than 6G bases of clean reads with a GC percentage above 47.19% and Q20 percentage above 95.16% after removing low quality reads and trimming the adapter sequences (Table 1). 

**Figure 2:**
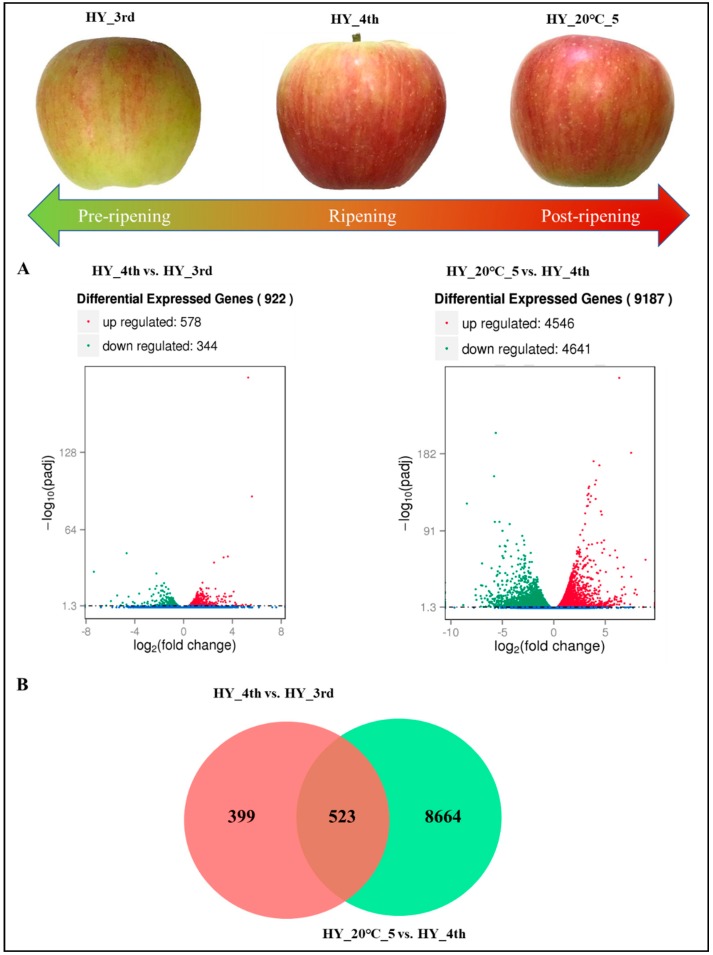
The number of differentially expressed genes (DEGs) within pre-(HY_4th vs. HY_3rd) and post-ripening (HY_20 °C_5 vs. HY_4th) groups (**A**). Overlapped DEGs were shown by Venn diagram between different groups (**B**).

To evaluate the significance of gene expression variation between pre- and post-ripening stages, the transcript abundance of each gene was normalized to the FPKM (Fragments Per Kilobase of transcript sequence per Millions of base pairs sequenced) value. The differentially expressed genes were identified and filtered for Q value <0.005 and log2Ratio >1. Total 922 unigenes were differentially expressed in pre-ripening (HY_4th vs. HY_3rd) samples where 578 genes were up-regulated and 344 genes were down-regulated between pre-ripening and ripening stages of apple fruit. The number of up-regulated and down-regulated unigenes is shown in the (Figure 2A, Table S1). Comparatively higher DEGs of 9187 unigenes were found in the post-ripening (HY_20 °C_5 vs. HY_4th) sample where 20 °C storage for 5 d up-regulated 4546 DEGs and downregulated 4641 genes (Figure 2A, Table S1). Overlapped DEGs between pre-and post-ripening groups are shown on a Venn diagram (Figure 2B).

### 2.3. GO Enrichment Classification and KEGG Pathway Analysis

**Figure 3:**
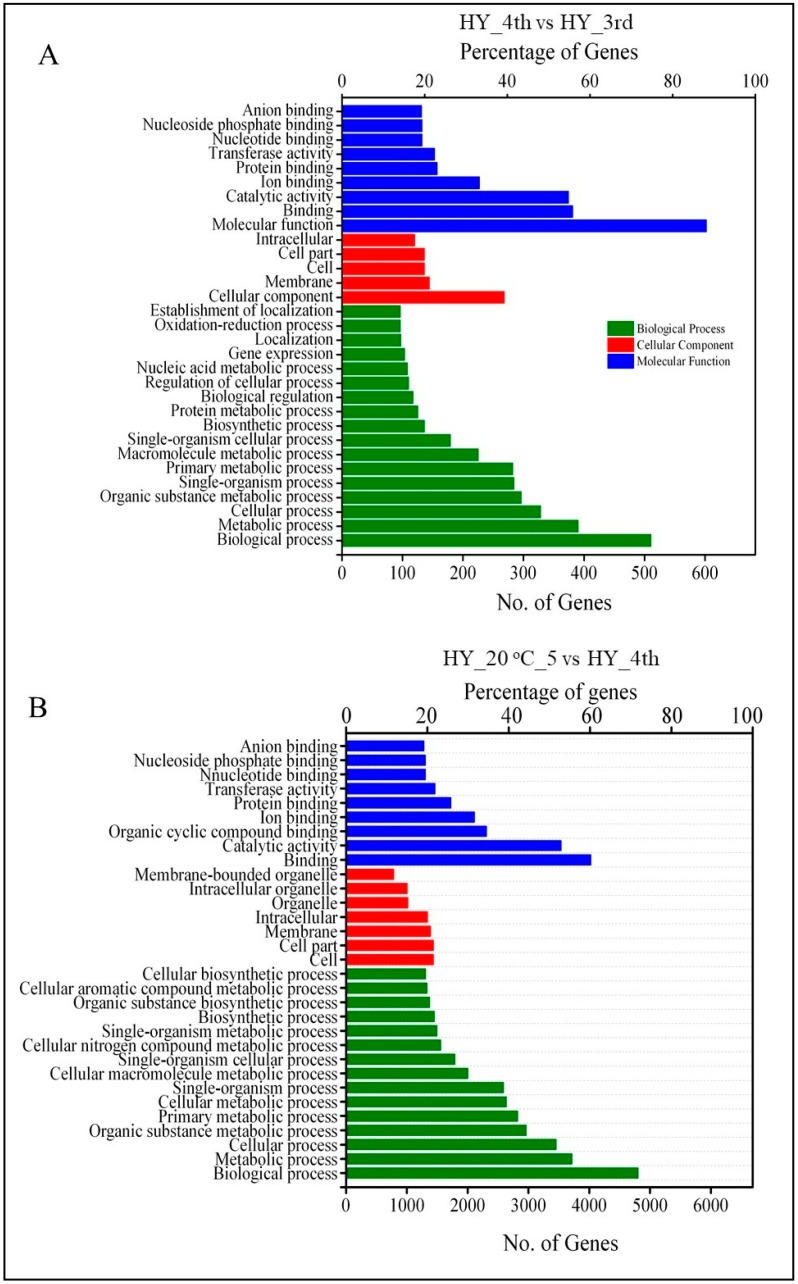
Classification of gene ontology (GO) terms concerning to differentially expressed genes in pre- (**A**) and post-ripening (**B**) apple fruit. The GO terms were classified into three categories, including molecular function, cellular component and biological process. Top 31 enriched GO terms were exhibited in each cluster.

Gene Ontology, an international standard classification system for gene function, was used to illustrate the role of DEGs in different groups. The DEGs were clustered into three main categories of the GO classification: Biological Process, Cellular Function and Molecular Function. In the pre-ripening group, 466 unigenes were assigned to the Biological Process category, 143 unigenes were assigned to the Cellular Component category and 351 unigenes were assigned to the Molecular Function category. These genes were further classified into 31 functional subcategories by mapped homology (Figure 3A, Table S2). The most common assignments in the Biological Process category were metabolic processes, cellular processes and organic substance metabolic processes. In the Cellular Component category, the majority of unigenes were grouped into the membrane, cell and cell part subcategories. Genes in the Molecular Function category were primarily sorted into the binding, catalytic activity and ion binding subcategories (Figure 3A, Table S2). In the post-ripening group, the most abundant GO terms in the Biological process include ‘metabolic process,’ ‘cellular process’ and ‘organic substance metabolic process.’ In the molecular function category, most of the DEGs were mapped onto the ‘binding,’ ‘catalytic activity’ and ‘organic cyclic compound binding’ groups. The cellular component category comprises the greatest numbers of genes in ‘cell,’ ‘cell part’ and ‘membrane’ groups (Figure 3B, Table S2).

**Figure 4:**
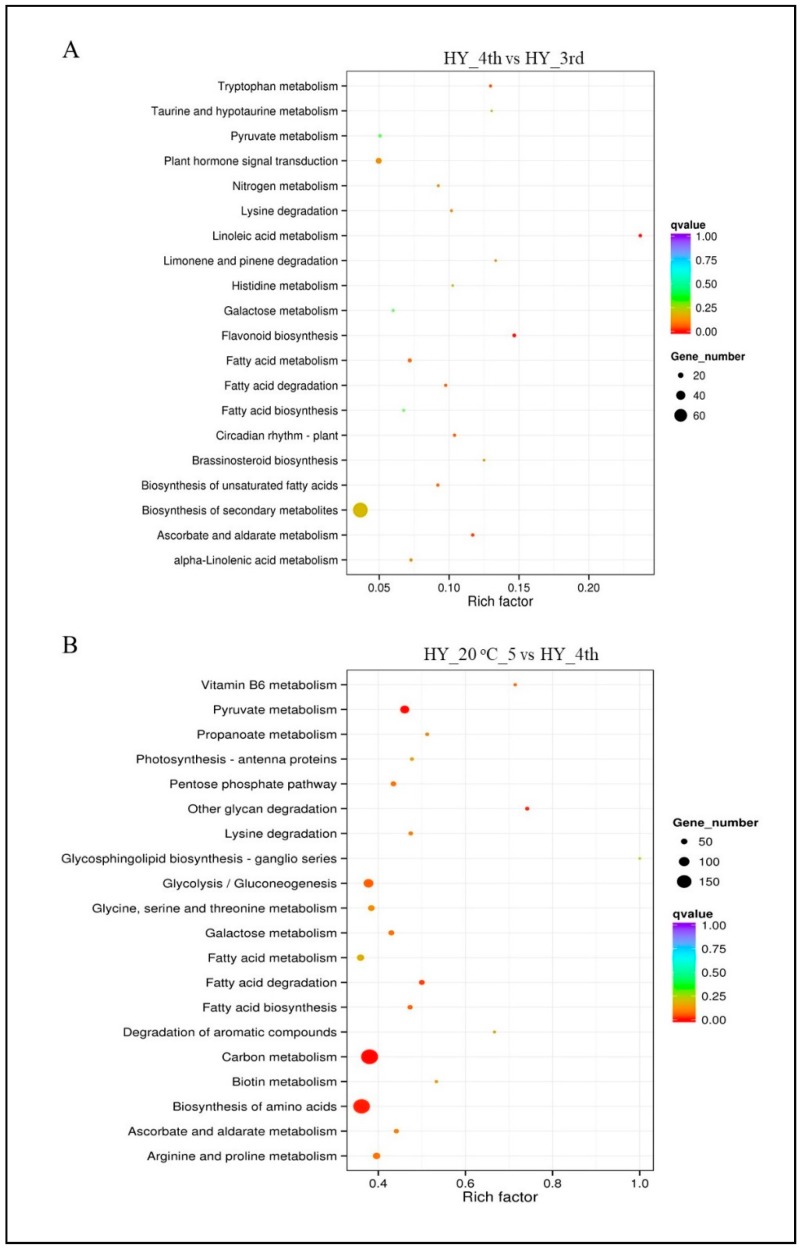
KEGG pathway enrichment concerning differently expressed genes in pre- (**A**) and post-ripening (**B**) apple. Top 20 enriched KEGG pathways were exhibited in each cluster. Rich factor is the ratio of DEGs counts to this pathway in the annotated genes counts. The more the q-value is close to zero, the more significant is the enrichment.

To identify the biological pathways activated in selected apple fruits, we mapped the annotated sequences to the reference pathways in the KEGG database [44]. In the pre-ripening samples, significant matches were found for 574 unigenes, which were assigned to 100 KEGG pathways (Figure 4A; Table S3). The KEGG pathway analysis has revealed the top 20 most enriched metabolic pathway subcategories, including the most abundant group represented as the ‘Biosynthesis of other secondary metabolites,’ ‘plant hormone signal transduction’ and ‘flavonoid biosynthesis.’ On the other hand, post-ripening samples exhibited the top 20 pathways where the largest groups fell into the ‘carbon metabolism,’ ‘biosynthesis of amino acids,’ ‘pyruvate metabolism,’ ‘glycolysis/gluconeogenesis’ and ‘arginine and proline metabolism’ categories (Figure 4B; Table S3). 

### 2.4. Confirmation of RNA-seq Results by qRT-PCR

**Figure 5:**
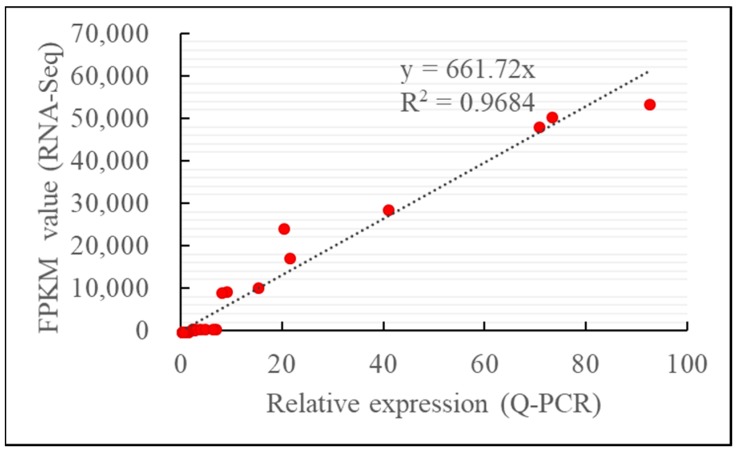
The correlation analysis of gene expression value from quantitative real-time PCR (Q-PCR) and RNA-Seq.

Genes showing differential expression involved in ethylene signaling pathway and anthocyanin biosynthesis in the transcriptome analysis were selected for qRT-PCR comparison of their expression levels in HY_3rd, HY_4th and HY_20 °C_5 samples, to confirm the accuracy and reproducibility of the transcriptome analysis. The scatter plot indicated higher integrity and correlation between transcriptome analysis through RNA-seq and qRT-PCR results (Figure 5). 

### 2.5. Identification of DEGs Related to Ripening Hormone

Plant hormones with diversified functions are obligatory for fruit development, maturation and ripening [6]. Several differentially expressed genes were identified as plant hormone regulators in our transcriptome study between pre- and post-ripening samples, which included ethylene, GA, auxin and ABA as well as BR (Table 2 and Table 3; Table S4). The most important hormone regulating fruit ripening is ethylene, which has been studied extensively. In the present study, the DEG analysis has revealed numerous regulatory genes which were directly associated with the ethylene signaling pathway. The post-ripening samples exhibited higher DEGs involved in the ethylene signaling pathway than pre-ripening samples (Table S4). Most of these genes, including ACO, EIN2, EIN3, ERS1, EIL and several ERFs showed higher expression in the post-ripening (Hy_20 °C_5) samples. Most of these were not expressed, or were expressed at a shallow level in the pre-ripening (HY_3rd) samples. This result showed consistency with the previous reports [7,45]. 

In addition to ethylene, other important plant hormones and the genes characterizing these hormones were also specified in the DEG analysis results. In the ABA biosynthesis pathway, the key catabolizing gene encoding *NCED1* (9-cis-epoxycarotenoid dioxygenase) was highly expressed in the post-ripening samples, suggesting possible regulation of ABA in the ripening of apple. Moreover, another gene involving in the ABA signaling, encoding Protein Phosphatase 2C, showed higher expression in the post-ripening samples and several genes encoding serine/threonine protein kinases were downregulated (Table 3; Table S4). 

**Figure 6:**
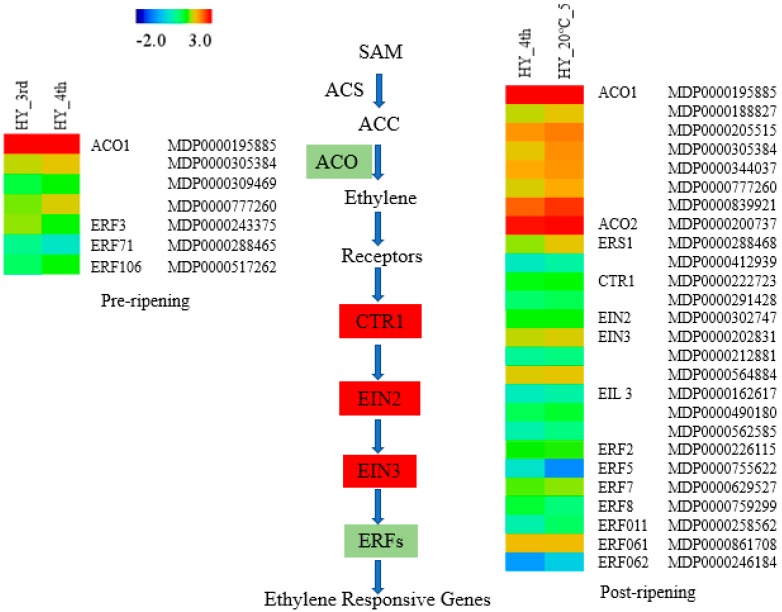
Heat map represents DEGs involved in ethylene signaling pathway in pre (HY_4th vs. HY_3rd) and post (HY_20 °C_5 vs. HY_4th) ripening groups. Genes remarked with light green color denotes expression in both groups and red color denotes expressed in the post-ripening group only. ACS (1-Aminocyclopropane-1-carboxylic acid synthase); ACO (1-Aminocyclopropane-1-carboxylic acid oxidase); ERS (Ethylene response sensor); CTR1 (CONSTITUTIVE TRIPLE RESPONSE 1); EIN2 (ETHYLENE INSENSITIVE 2); EIN3 (ETHYLENE INSENSITIVE 3); EIL3 (ETHYLENE INSENSITIVE 3-like 3); and ERFs (Ethylene Response Factors).

Previous studies have stated that conjugation of IAA-Asp was primarily regulated by the association of GH3 (indole-3-acetic acid amino synthase) gene in the auxin signaling pathway [10]. The up-regulation of this gene in post-ripening samples indicates its role in decreasing free IAA during fruit ripening (Table 3; Table S4). Furthermore, up- or down-regulation of several auxin response factors (ARF) were observed in the post-ripening samples, which may have functional regulation and play different roles in fruit ripening (Table 3; Table S4). The inactivation of GA controlled by a primary gene encoding gibberellin 2-beta-dioxygenase (GA2ox1) was observed to be highly expressed in the post-ripening samples (Table 3; Table S4). In the BR pathway, several genes encoding cytochrome p450 were downregulated in the post-ripening samples and a lower expression of other genes in the BR pathway indicates inferior transcript abundance of these genes, representing its negative regulation of the fruit ripening process (Table 3; Table S4). A heat map including all the regulating genes involved in the ethylene signaling pathway are shown in Figure 6.

### 2.6. DEGs Involved in Anthocyanin Biosynthesis

‘Hongyu’ apple fruits undergo a progressive color alteration to red at the later stages of ripening. Anthocyanin most prominently changed the red color of apple fruit, comprising mostly of cyanidin 3-galactoside [46]. Studies with numerous crops, including pear and grape, have specified an imperative correlation of anthocyanin content with the expression of its biosynthetic genes [47,48]. Several DEGs related to flavonoid biosynthesis were identified in the KEGG pathway assessment, which has exposed the differential expression of the genes such as *PAL*, *4CL*, *CHI*, *F3H*, *DFR* and *UFGT* involved in the anthocyanin biosynthesis pathway in post-ripening samples. In contrast, most were not expressed or were expressed at a low level in pre-ripening samples. In this study, 20 candidate genes that are responsible for anthocyanin synthesis in fruit were identified from the DEG data including *PAL* (2 unigene), *4CL* (3 unigenes), *CHI* (3 unigene), *F3H* (5 unigene), *DFR* (4 unigene) and *UFGT* (3 unigenes) in post-ripening samples (Table S5). On the other hand, genes encoding *PAL* (2 unigenes), *F3H* (4 unigenes) and *CHI* (4 unigenes) were differentially expressed in pre-ripening samples. All of the characterized DEGs regarding anthocyanin biosynthesis showed higher expression in the post-ripening samples (Table S5). A previous qRT-PCR study of the genes associated with the anthocyanin biosynthesis process also showed similar results [49]. The genes regulating the last steps of anthocyanin biosynthesis encoding *UFGT* was also highly expressed in the post-ripening samples. This result indicates that *UFGT* may play a key role in apple fruit coloration during ripening. 

**Figure 7:**
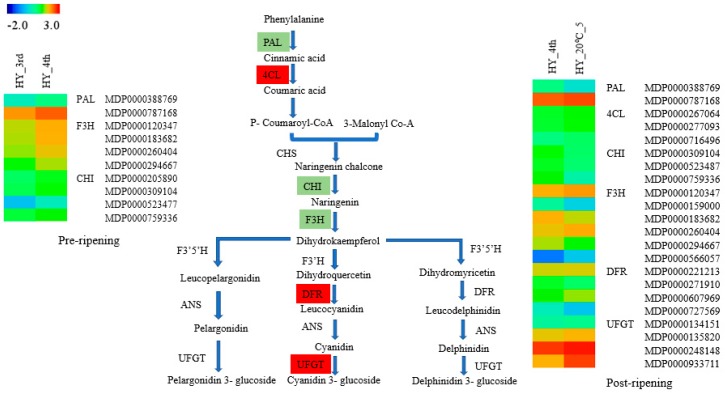
Heat map represents DEGs involved in anthocyanin biosynthesis pathway in pre (HY_4th vs. HY_3rd) and post (HY_20 °C_5 vs. HY_4th) ripening groups. Genes remarked with light green color denotes expression in both groups and red color denotes expressed in the post-ripening group only. PAL (Phenylalanine ammonia-lyase); 4CL (4-coumarate-CoA ligase); CHS (Chalcone Synthase); CHI (Chalcone isomerase); F3H (Flavanone 3-hydroxylase); F3′H (Flavanone 3′-hydroxylase); DFR (Dihydroflavonol 4-reductase); ANS (Anthocyanidin synthase) and UFGT (UDP glucose: flavonol 3-O-glucosyltransferase).

A heat map involving the DEGs expressed at pre-and post-ripening apple fruit in anthocyanin biosynthesis process shown in Figure 7. The biosynthesis and accumulation of anthocyanin are primarily responsible for fruit coloration which might be controlled by the expression of these genes. 

### 2.7. Transcriptional Regulation during Fruit Ripening

To date, many transcription factors have been specified to regulate fruit ripening processes [50]. A well-known transcription factor is the *RIN* gene, a MADS-box gene family. In our study, numerous transcription factors including the MADS-box gene, *MYB*, *bHLH*, *NAC*, *WRKY*, *HSF* were identified in the DEG analysis data between pre- and post-ripening samples. In the post-ripening samples, one MADS-box gene AGL8 was downregulated whereas the other two genes AGL15 and AGL80 were upregulated. A RIN-homologue of apple MADS8/9 has been found to regulate fruit ripening by directly controlling the auxin levels [51]. Mature MADS8/9-suppressed apples demonstrated reduced expression of GH3 and exhibited a higher concentration of free IAA [51]. In our results, the higher expression of two MADS-box genes and consequently up-regulation of *GH3* genes were observed in post-ripening samples. Moreover, six *MYB* genes and five *NAC* genes were upregulated in the post-ripening samples. On the other hand, stress-related protein *HSF* also showed differential expression (up or down) in the post-ripening samples (Table S6). According to the results, several other *bHLH* transcription factors were expressed to be up or down-regulated. The differential expression of these regulatory transcription factors may control hormonal regulation and anthocyanin biosynthesis during the ripening process. 

## 3. Discussion

Fruit ripening is considered to be a crucial parameter that influences the shelf life of fruit and its overall market value. The ripening process involves the initiation of multiple gene expression and biochemical pathways. However, the underlying molecular mechanism of their regulators remains to be uncovered. A deeper understanding of hormonal regulation and anthocyanin biosynthesis in the fruit ripening process provides the potential to investigate post-harvest management and fruit quality. In this study, we compared the fruit transcriptomes in pre- and post-ripening fruits of an early ripening apple cultivar and found that 9187 genes were differentially expressed after post-harvest storage for 5 d at 20 °C. Several DEGs were included in the GO terms involved in the biosynthesis and regulation of plant hormones including ethylene and the enrichment of the flavonoid biosynthesis pathway in the KEGG analysis.

Ethylene is supposed to be critical for controlling the ripening of climacteric fruits [52]. As an important ripening hormone, various studies on model climacterics fruits, such as the tomato and the banana, have focused on genes involved in the biosynthesis and signal transduction of ethylene in ripening [45,53]. In the present study, transcriptome analysis with the fruit prior to, and immediately after, ripening exposed large numbers of DEGs related to ripening. Using microarray analysis, 106 genes were found to be highly coordinated with fruit ripening in the tree ripening of the ‘Royal Gala’ apple [54]. DEGs specified in the ethylene and another hormone signaling process were mostly expressed in the post-ripening (HY_20 °C_5) samples. ABA is an important plant hormone, which has been anticipated to play vital roles in the ripening processes of both climacteric and non-climacteric fruits [46,55]. ABA promotes ripening by promoting ethylene biosynthesis through the up-regulation of ethylene biosynthesis genes [22]. It has been considered a ripening-inducer in strawberry and grapefruits [55,56]. The expression of the key gene for ABA biosynthesis encoding *NCED1* increases at the breaker stage of tomato fruits and suggests an important role for ABA in triggering ethylene biosynthesis [39]. Furthermore, a longer shelf life of tomato fruits was observed due to the down-regulation of some ripening-related cell wall genes mediated by the suppression of *LeNCED1* [22]. Similarly, the reduction of *NCED* expression was correlated with retardation of ripening in the strawberry [55]. In our results, genes encoding *NCED1* were up-regulated in the post-ripening samples and showed consistency with previous studies. In another study, the application of 1-MCP on ‘nanguo’ pear fruits extended the shelf life by subsequently suppressing the expression of the *NCED1* gene [57]. The expression pattern of *NCED1,* which directly correlated with ABA levels, was observed in tomato (*SlNCED1*), persimmon (*DkNCED1*), sweet cherry (*PacNCED1*) [58] and cucumber (*CsNCED1*), which declined rapidly during the postharvest stage [22]. These findings also provide substantial evidence of ABA as critical and affects the regulation of fruit ripening. Another most shared plant hormone is auxin, found as a free form of IAA (indole-3-acetic acid IAA) and reported to decline in concentration prior to fruit ripening [12,59]. However, conjugation of IAA and the formation of IAA-Asp (IAA-aspartic acid), mediated by GH3 (IAA-amido synthetase,) increased in ripened fruit [13]. Similarly, the up-regulation of *GH3.1*, *GH3.5*, *GH3.9* and *GH3.17* in the strawberry seed tissues with high auxin levels indicated that even IAA biosynthesis might induce IAA conjugation by promoting the expression of members of this gene family [60]. Furthermore, a higher expression of GH3 and subsequent delays of fruit ripening with IAA treatment were observed in the onset of the ripening of grape berries [13]. Our results also supported this where GH3 was up-regulated in the post-ripening samples. These findings directed the functional roles of GH3 in reducing the IAA level and promoting fruit ripening. Studies with the regulation of auxin in transcript level indicated that an increase in firmness and prolonged shelf life was mediated by the down-regulation of the *ARF4* gene in the tomato [61]. In our results, we also found that *ARF* genes were downregulated in the post-ripening samples, suggesting a role in fruit ripening. 

From our DEG analysis results, we also found several key genes involved in the anthocyanin biosynthesis pathway, including *PAL*, *4CL*, *CHI*, *F3H* and *UFGT*. The distinct functions of these genes have been characterized in previous studies. In a study with a green and red apple cultivar, it was observed that the genes involved in the anthocyanin biosynthesis pathway such as *CHS*, *F3H*, *DFR* and *UFGT*, were expressed at a very low level in green apple varieties, where the expression of these genes was remarkably higher in red apple cultivar [62]. Our results showed a higher expression of *PAL*, *4CL*, *DFR*, *F3H* and *UFGT* genes in the post-ripening samples. A previous qRT-PCR study showed an up-regulation of these genes along with higher anthocyanin levels and ripening progress [49]. The expression of UFGT was investigated in different fruits like grape, strawberry and lychee and was found to be critical in controlling fruit color development [63]. Also, the involvement of transcription factors regulating the structure genes expression also demonstrated and suggested a key role of *MYB* and the basic-helix-loop-helix (*bHLH*) families’ genes on the regulation of anthocyanin biosynthesis related genes [64]. Another study also reported that *MYB* transcription factors might play a key role in regulating anthocyanin biosynthesis in some fruit [65,66]. In our study, several *MYB* and *bHLH,* including other genes, were differentially expressed in the post-ripening samples. Thus, these transcription factors may regulate the expression of key structure genes associated with the anthocyanin biosynthesis pathway. 

However, future studies on critical observations of the changes of these regulatory factors are crucial. Furthermore, the association and crosstalk between the differentially expressed structure genes and corresponding transcription factors in hormonal regulation and anthocyanin biosynthesis during ripening are still indistinct and this suggests further investigation.

## 4. Materials and Methods

### 4.1. Plant Material

‘Hongyu’ apples (*Malus domestica* L. Borkh) were used in this study. Fruits of uniform size and color at two different developmental stages of 110 days after flowering (DAF) and 120 DAF were collected from an orchard in Changping District, Beijing, China. Another group of fruits harvested at 120 DAF (considered as commercial maturity) was allowed to ripen for 5 d at 20 °C. Fruit at 120 DAF was considered to be commercially ripened according to the defined harvesting date by the particular apple orchard. To investigate the sudden hormonal changes and anthocyanin biosynthesis during ripening, a sampling date was selected before commercial ripening (110 DAF) and immediately after harvesting by allowing the fruits to be stored for 5 d at 20 °C. The environmental conditions and cultural practices were maintained according to standard apple orchard practices. Fruits at each sampling point were transported to the laboratory as soon as possible, the flesh was taken and immediately frozen in liquid nitrogen and stored at −80 °C for further use. Each sample consisted of nine randomly selected fruits, separated into three replicates with three fruits in each. The fruits of 110 DAF, 120 DAF and stored at 20 °C for 5 d were considered for RNA-seq analysis and assigned to a pre-ripening and a post-ripening group. 

### 4.2. Organic Acids and Sugars Measurement

Organic acid and sugar content was measured by following a modified method previously described by Lin et al. [67]. Briefly, a total of 1 g sample powder was homogenized with 4 mL of 80% ethanol. The mixture was extracted with ultra-sonication for 30 min at room temperature and centrifuged at 10,000 *g* for 15 min. Aliquots of 1 mL of the upper phase were dried with pure nitrogen. The residue was dissolved in 1 mL ddH_2_O and filtered through a membrane with 0.22 μm pore size. For sugar analysis, a volume of 10 μL for each sample was injected into the ion chromatograph (ICS-3000, Dionex, Sunnyvale, CA, USA) fitted with Carbo PacTMPA20 column (3 mm × 150 mm). The column temperature was 35 °C and the flow rate was 0.5 mL min^−1^. The gradient elution buffer was used as follows: A, ddH_2_O; B, 250 mmol L^−1^ NaOH; equal gradients of 92.5% A and 7.5% B were used for elution. A pulsed amperometric detector with gold electrode was used. For organic acid analysis, a volume of 25 μL for each sample was injected into the ion chromatograph (ICS-3000, Dionex, Sunnyvale, CA, USA) fitted with IonPac AS11-HC column (4 mm × 250 mm). The column temperature was 30 °C and the flow rate was 1 mL min^−1^. The gradient elution procedure was 0.8 mmol L^−1^ KOH, 0–12 min; 0.8–34 mmol L^−1^ KOH, 12–40 min; 34 mmol L^−1^ KOH, 40–50 min. The electrical conductivity detector was used for detection. Substances were identified according to the retention time (RT) of standard compounds. The contents of glucose, fructose, sucrose, malate and oxalate were calculated by comparison with standard curves.

### 4.3. Total RNA Extraction

Total RNA was extracted using a modified CTAB method [68]. RNA quantity and quality (purity) were analyzed using a UV-Vis spectrophotometer (Merinton Instrument, Inc., Ann Arbor, MI, USA).

### 4.4. Library Construction

Total RNA from 2 different fruit developmental stages (pre-ripening and ripening) and post-harvest stored apples at 20 °C (post-ripening) were pooled before library preparation and an equimolar quantity of total RNA from samples at each stage were combined into one pool. Prior to cDNA library construction, poly-T oligo-attached magnetic beads were used to purify the mRNA, which was added to a fragmentation buffer to break mRNA into short segments of approximately 200 bp followed by cDNA synthesis using random hexamers and reverse transcriptase. Then buffer, dNTPs, RNase H and DNA polymerase I were added to synthesize the second strand cDNA. Both ends of sequencing adapters were ligated after terminal repairing of double-stranded cDNA fragments. The AMPure XP system (Beckman Coulter, Beverly, MA, USA) was used to purify the final cDNA library and selectively enriched by PCR enrichment. The library preparations were sequenced by Novogene Bioinformatics Technology Co., Ltd. (Beijing, China) on an Illumina HiSeq^TM^ PE150 platform. Three replications were used in each group for the precision of results. Fruit of 110 DAF denoted as HY_3rd, 120 DAF indicated as HY_4th and ripened for 5 d at 20 °C were designated as HY_20 °C_5 groups in transcriptome analysis. 

### 4.5. Functional Annotation and Analysis of Differential Gene Expression

Raw reads were cleaned by removing low-quality reads and adapter sequence reads and clean reads were mapped to the apple reference genome (https://www.rosaceae.org/organism/Malus/x-domestica) using TopHat software [69]. The analysis of differentially expressed genes (DEGs) was done using a method previously reported in Reference [70] and *p*-value thresholds were measured by the false discovery rate (FDR) determination via multiple testing [71]. For gene expression quantification, the read numbers mapped to each gene were counted using HTSeq v0.6.1 and then normalized to FPKM (Fragments per Kilobase of transcript sequence per Millions of base pairs sequenced) [70]. For analysis of differential expression of the unigenes between two samples, the DESeq package (v1.10.1) was used and a corrected P-value threshold of 0.005 (using the Benjamini & Hochberg method) and log2 (fold change) of 1 were considered for filtering the DEGs value. For Gene ontology (GO) enrichment analysis of differentially expressed genes, GOseq (v2.12) was used [72]. KEGG enrichment analysis was performed using the KOBAS (v 2.0) software (Center for Bioinformatics, Peking University, Beijing, China) [73].

### 4.6. Quantitative Real-Time PCR (qRT-PCR)

First strand cDNA was synthesized from 1 μg RNA, after digestion of genomic DNA, using the iScriptTM cDNA Synthesis Kit (Bio-Rad, Hercules, California, USA). qRT-PCR was performed using the Power SYBR^®^ Green PCR Master Mix kit (Applied Biosystems, Foster City, CA, USA) on an ABI 7500 instrument (Applied Biosystems, Thermo Fisher Scientific, Waltham, MA, USA), initiated by 5 min at 95 °C then followed by 45 cycles of 95 °C for 10 s, 60 °C for 10 s and 72 °C for 15 s and completed with a melting curve analysis program. No-template controls and melting curve analyses were included in every reaction. The actin gene was included as an internal control. The comparative CT method (2^−ΔΔCT^ method) was used to analyze the expression levels of the different genes [74]. Primers used in this analysis are listed in Table S7.

### 4.7. Statistical Analysis

Figures were drawn using Origin 8.6 (Microcal Software Inc., Northampton, MA, USA). The MultiExperiment Viewer software (MeV v4.8.1, J. Craig Venter Institute, La Jolla, CA, USA) was used to represent the relative values of gene expressions.

## 5. Conclusions

The present study investigated the transcriptome profiles of an early ripening apple cultivar prior to, and immediately after, the commercial ripening stage using DGE deep-sequencing technologies through Illumina RNA-seq. This transcriptome analysis revealed that, in addition to ethylene biosynthesis genes, key genes of ABA biosynthesis (*NCED1*) and several auxin response factors were differentially expressed in the post-ripening samples. This result suggested that investigating the interplay of different hormones and respective genes that are differentially expressed in post-harvest fruit at the commercial maturity stage will be helpful for discovering the possible hormonal crosstalk in ripening regulation and for extending the ripening period of early ripening apples. This study also focused on apple fruits upon ripening, which also suggests that the up-regulation of DEGs involved in anthocyanin biosynthesis plays an important role in the accumulation of anthocyanin, similar to the results of previous studies on many fruits. The genes encoding *F3H* and *UFGT* were significantly upregulated in the post-harvest fruit, indicating key roles in anthocyanin biosynthesis. Several transcription factors were also specified including MADS-box genes, *MYB*, *WRKY*, *NAC*, *bHLH* and *HSF*, which may regulate the ripening process by the regulation of ethylene and other hormones and anthocyanin biosynthesis. 

## Figures and Tables

**Table 1:** Statistics of deferential gene expression (DGE) library sequencing and read mapping.

Groups	Library	Clean Bases	Error Rate (%)	Q20 (%)	GC Content (%)	Total Mapped Reads (%)	Uniquely Mapped Reads (%)
HY_3rd	HY001	8.03G	0.03	95.16	47.19	90.58	75.78
	HY002	6.04G	0.02	97.11	47.26	93.64	78.28
	HY003	7.15G	0.02	96.31	47.31	91.92	76.86
HY_4th	HY004	7.97G	0.03	94.98	47.47	90.54	75.68
	HY005	9.56G	0.02	97.00	47.58	92.33	76.47
	HY006	7.16G	0.03	95.05	47.37	90.99	76.13
HY_20 °C_5	HY007	7.42G	0.02	95.61	47.40	89.88	75.53
	HY008	6.85G	0.02	95.58	47.48	89.81	75.41
	HY009	7.67G	0.02	95.64	47.56	90.17	75.63

**Table 2:** DEGs related to plant hormones expressed in pre-ripening (HY_4th vs. HY_3rd) group.

DEGs	Log2Fold Change	P (adj)	Regulation	Annotation
Abscisic acid				
MDP0000437033	1.8445	0.027924	up	ARATH Protein phosphatase 2C 56 *Arabidopsis thaliana*
MDP0000929213	−0.75936	0.017195	down	9-cis-epoxycarotenoid dioxygenase NCED1 *Phaseolus vulgaris*
MDP0000165867	0.60882	0.013085	up	Serine/threonine-protein kinase EDR1 *Arabidopsis thaliana*
MDP0000226571	−0.7009	0.027422	down	Serine/threonine-protein kinase HT1 *Arabidopsis thaliana*
Auxin				
MDP0000132805	−0.79202	7.43 × 10^−5^	down	Auxin-responsive protein IAA13 *Arabidopsis thaliana*
MDP0000268306	0.54285	0.042346	up	Auxin response factor 6 *Arabidopsis thaliana*
MDP0000137461	0.69595	0.030746	up	Auxin response factor 8 *Arabidopsis thaliana*
MDP0000666539	3.2911	7.27 × 10^−10^	up	Indole-3-acetic acid-amido synthetase GH3.3 *Arabidopsis thaliana*
Gibberellin				
MDP0000301368	1.1041	0.010819	up	Scarecrow-like protein 3 *Arabidopsis thaliana*
MDP0000929994	0.71505	8.31 × 10^−5^	up	Gibberellin receptor GID1B *Arabidopsis thaliana*
Brassinosteroid				
MDP0000261851	1.4218	0.016674	up	G-type lectin S-receptor-like serine/threonine-protein kinase At4g27290 *Arabidopsis thaliana*
MDP0000361876	−2.9427	2.40 × 10^−5^	down	Xyloglucan endotransglucosylase/hydrolase protein 9 *Arabidopsis thaliana*
MDP0000120044	1.0382	3.17 × 10^−5^	up	Cytochrome P450 714A1 *Arabidopsis thaliana*
MDP0000130467	−0.63388	0.017284	down	Cyclin-U4-1 *Arabidopsis thaliana*

**Table 3:** DEGs related to plant hormones expressed in post-ripening (HY_20 °C_5 vs. HY_4th) group.

DEGs	Log2Fold Change	P (adj)	Regulation	Annotation
Abscisic acid				
MDP0000929213	1.9744	3.68 × 10^−46^	up	9-cis-epoxycarotenoid dioxygenase NCED1, *Phaseolus vulgaris*
MDP0000174607	0.84671	0.000358	up	Protein phosphatase 2C 57 *Arabidopsis thaliana*
MDP0000165966	−0.6786	0.0035436	down	Serine/threonine-protein kinase HT1 *Arabidopsis thaliana*
MDP0000232165	−0.7273	9.29 × 10^−5^	down	Serine/threonine-protein kinase ATM *Arabidopsis thaliana*
Auxin				
MDP0000209432	2.2878	3.71 × 10^−41^	up	Probable indole-3-acetic acid-amido synthetase GH3.1 *Arabidopsis thaliana*
MDP0000153538	−1.1354	0.0022471	down	Auxin response factor 6 *Arabidopsis thaliana*
MDP0000246042	1.8749	5.27 × 10^−7^	up	Auxin-responsive protein IAA31 *Arabidopsis thaliana*
MDP0000876321	−2.0633	2.73 × 10^−9^	down	Auxin response factor 19 *Arabidopsis thaliana*
Gibberellin				
MDP0000137705	6.5988	5.24 × 10^−23^	up	Gibberellin 2-beta-dioxygenase 1 *Pisum sativum*
MDP0000319522	0.7380	0.000418	up	Gibberellin receptor GID1B *Arabidopsis thaliana*
MDP0000901967	−5.0642	3.65 × 10^−14^	down	Gibberellin-regulated protein 14 *Arabidopsis thaliana*
MDP0000119093	1.4537	1.64 × 10^−21^	up	Scarecrow-like protein 9 *Arabidopsis thaliana*
MDP0000256486	−1.3716	0.0014764	down	Scarecrow-like protein 4 *Arabidopsis thaliana*
Brassinosteroid				
MDP0000210409	−2.6398	1.74 × 10^−7^	down	G-type lectin S-receptor-like serine/threonine-protein kinase B120 *Arabidopsis thaliana*
MDP0000140341	−2.1661	2.10 × 10^−12^	down	LRR receptor-like serine/threonine-protein kinase RCH1 *Arabidopsis thaliana*
MDP0000183140	−1.3888	0.015187	down	Xyloglucan glycosyltransferase 4 *Arabidopsis thaliana*
MDP0000127773	−1.0592	7.65 × 10^−6^	down	Cytochrome P450 90A1 *Arabidopsis thaliana*
MDP0000140803	0.6914	0.036655	up	Cytochrome P450 78A6 *Arabidopsis thaliana*
MDP0000154776	−2.3984	4.95 × 10^−6^	down	Cytochrome P450 734A1 *Arabidopsis thaliana*
MDP0000176105	−1.6388	0.011614	down	Cyclin-D4-1 *Arabidopsis thaliana*

## Supplementary Materials

The following are available online, Table S1: List of annotated DEGS, Table S2: List of GO enrichment analysis results, Table S3: List of KEGG pathway enrichment analysis results, Table S4: List of DEGs related to plant hormone signaling pathway between pre and post-ripening samples, Table S5: List of DEGs involved in anthocyanin biosynthesis pathway, Table S6 List of DEGs related to different transcription factors, Table S7: List of Primers used for gene expression analysis.
