# Supplementary material for: Comparative Transcriptomic Profiling to Understand Pre- and Post-Ripening Hormonal Regulations and Anthocyanin Biosynthesis in Early Ripening Apple Fruit
Source: Molecules. 2018 Jul 31;23(8):1908. doi: 10.3390/molecules23081908 (PMC6222687; doi:10.3390/molecules23081908)
Supplement: Supplementary file 1 [file molecules-23-01908-s001.zip › Table S7 List of Primers used for gene expression analysis.docx]

Table S7. List of primers for qRT-PCR analysis.

| **Genes** | **Primers** | **Sequences (5' -3')** | **Access No.** |
| --- | --- | --- | --- |
| *MdACO1* | ACO1-F | ATCAATGATGCTTGTGAGAACTG | MDP0000195885 |
|  | ACO1-R | GGTCTTCTTGTAGTGATCCTTGG |  |
| *MdACO2* | ACO2-F | TCGGACGGAACCAGAATG | MDP0000200737 |
|  | ACO2-R | CTCCTTGGCTTGGAATTTGA |  |
| *MdERS1* | ERS1-F | TCCAGAACTGGTATGAACCTACA | MDP0000412939 |
|  | ERS1-R | AGAACTGTTGAAGACTTCGTTGA |  |
| *MdCTR1* | CTR1-F | ACAAGATTTTCATGCCGAAC | MDP0000291428 |
|  | CTR1-R | TATGGACAAGTTTGGAGGCT |  |
| *MdEIN2* | EIN2-F | GAGCCGCAGTACCATTCTTC | MDP0000302747 |
|  | EIN2-R | CCTGAAGCCGGTTGAGAAC |  |
| *MdEIL3* | EIL3-F | CCCAAGTCAAGACCAGCAG | MDP0000562585 |
|  | EIL3-R | CTCCAGCAATCTCACTTCCA |  |
| *MdActin* | Actin-F | TGACCGAATGAGCAAGGAAATTACT | CN938023 |
|  | Actin-R | TACTCAGCTTTGGCAATCCACATC |  |
